# Experimental Study on Machinability of Zr-Based Bulk Metallic Glass during Micro Milling

**DOI:** 10.3390/mi11010086

**Published:** 2020-01-13

**Authors:** Tao Wang, Xiaoyu Wu, Guoqing Zhang, Bin Xu, Yinghua Chen, Shuangchen Ruan

**Affiliations:** 1 Guangdong Provincial Key Laboratory of Micro/Nano Optomechatronics Engineering, College of Mechatronics and Control Engineering, Shenzhen University, Nan-hai Ave. 3688, Shenzhen 518060, China; tao.bit2010@hotmail.com (T.W.); zhanggq@szu.edu.cn (G.Z.); xubin_szu@163.com (B.X.); 2172291743@email.szu.edu.cn (Y.C.); 2 Key Laboratory of Optoelectronic Devices and Systems of Ministry of Education and Guangdong Province, College of Optoelectronic Engineering, Shenzhen University, Shenzhen 518060, China; scruan@szu.edu.cn

**Keywords:** micro milling, bulk metallic glass, surface roughness, milling force, tool wear

## Abstract

The micro machinability of Zr_41.2_Ti_13.8_Cu_12.5_Ni_10_Be_22.5_ bulk metallic glass (BMG) was investigated by micro milling with coated cemented carbide tools. The corresponding micro milling tests on Al6061 were conducted for comparison. The results showed that the tool was still in stable wear stage after milling 300 mm, and the surface roughness Ra could be maintained around 0.06 μm. The tool experienced only slight chipping and rubbing wear after milling the BMG, while a built-up edge and the coating peeling off occurred severely when milling Al6061. The influence of rotation speed on surface roughness was insignificant, while surface roughness decreased with the reduction of feed rate, and then increased dramatically when the feed rate was below 2 μm/tooth. The surface roughness increased gradually with the axial depth of cut (DOC). Milling force decreased slightly with the increase in rotation speed, while it increased with the increase in axial DOC, and the size effect on milling force occurred when the feed rate decreased below 1 μm/tooth. The results of X-ray diffraction (XRD) showed that all milled surfaces were still dominated by an amorphous structure. This study could pave a solid foundation for structural and functional applications.

## 1. Introduction

Due to the lack of a long-range ordered atomic structure, metallic glasses (also called amorphous alloys) generally do not have crystallites, grain boundaries, and dislocations. The special structure and metastable state provide unusual properties, including superior strength, hardness, and elastic strain limit, as well as excellent corrosion and wear resistance [1]. They drew great attention in both academic and industrial fields due to their structural and functional applications [2].

To achieve high dimensional accuracy and excellent surface quality for practical applications, cutting is one of the most fundamental processes for shaping bulk metallic glass (BMG) parts. Bakkal et al. [3,4,5,6,7] comprehensively studied the macro machinability of a Zr_52.5_Ti_5_Cu_17.9_Ni_14.6_Al_10_ metallic glass by turning, drilling, and milling comprehensively, and found that the generated heat in the cutting process could cause the oxidation and crystallization of chips, and severe tool wear. Shear bands, void formation, and viscous flow were observed in chip morphology. Jiang and Dai [8] proposed that the underlying formation mechanism of lamellar chips in cutting Vit 1 bulk metallic glass was the symmetry breaking of free volume flow and source, rather than thermal instability. Fujita et al. [9] investigated the cutting characteristics of both Zr_65_Cu_15_Ni_10_Al_10_ and Pd_40_Cu_30_Ni_10_P_20_ BMGs by turning, and attributed the excellent cuttability of BMGs to a slipping-off mechanism at planes of very short intervals determined only by the maximum shear stress. Furthermore, non-built-up edges were observed. Yan et al. [10] developed an analytical model based on the Mohr–Coulomb criterion for cutting metallic glasses, and the model was verified by comparing the predicted cutting forces with the measured forces. Chen et al. [11] investigated the micro machinability of Zr_55_Cu_30_Al_10_Ni_5_ bulk metallic glass using an ultra-precision cutting process. The results indicated that the metallic glass could only attain the lowest surface roughness of about Ra 100 nm, and achieving a mirror surface was hard due to its viscous flow. Dhale et al. [12] studied the chip formation process in orthogonal cutting of Zr-based bulk metallic glass, and found that serrated structures could be observed at all the cutting speeds investigated. Secondary shear bands occurred at the individual serrated region at low cutting speeds, while fracture and fragmentation at the individual serrated region were observed at high cutting speed. Maroju and Jin [13] established a physical model to study the fundamental mechanism of segmented chip formation in orthogonal cutting of Zr-based BMG. The results indicated that the shear stress oscillations in the primary shear zone are initiated by the increase in free volume. Moreover, the temperature in the PSZ (primary shear zone) plays a significant role in the estimation of the frequency of chip segmentation.

Micro milling is recognized as one of the most versatile machining processes to fabricate micro components and micro features [14], while few papers reported micro milling of BMG materials. In the micro milling process, there are many phenomena affecting the chip formation, cutting force, and surface profile constitution. Combining the plastic formation theory with the slip line field theory, Wan et al. [15] proposed a material separation model to theoretically calculate the shape of the dead metal zone, and the minimum uncut chip thickness (MUCT) could be derived. Yuan et al. [16] proposed an innovative uncut chip thickness algorithm which could consider the effects of the excat trochoidal trajectory of the tool tip and the cutting trajectory of all previously passing teeth, tool runout, minimum chip thickness, material elastic recovery, and the variation of the entry and exit angles. Wojciechowski et al. [17] proposed an original force model considering the effects of micro end milling kinematics, geometric errors of the machine tool–toolholder–mill system, elastic and plastic deformations of workpiece correlated with the minimum uncut chip thickness, flexibility of the slender micro end mill, and the chip thickness accumulation phenomenon. Wojciechowski and Mrozek [18] evaluated the influence of tool axis inclination angle on the dynamics of micro ball end milling, and found that the decrease in tool axis inclination caused the nonlinear growth of vibration amplitudes and surface roughness *R*_t_ parameter values. Wojciechowski [19] established a surface roughness model including cutter displacements in the cylindrical milling process, and found that real roughness parameters were significantly higher than those calculated on the basis of a kinematic–geometric basic model; their values were strongly dependent on dynamic cutter displacements. 

The above-discussed studies provided insight into the main phenomena in the micro milling process of conventional crystalline metal, while few papers reported micro milling of BMG materials. Maroju et al. [20] investigated surface microstructure in high-speed milling of Zr-based bulk metallic glass, and they found that the crystallization of originally amorphous BMG material occurred. However, the diameter of their milling tool was 3.175 mm, which is still in the range of macro milling.

This paper presents an experimental study on machinability and surface integrity during the micro milling of Zr-based bulk metallic glass, which could pave a solid foundation for its applications in the future, e.g., the mold for microfluidic devices.

The objectives of this paper were as follows:

1. Comparative study of the tool wear mechanism and its influence on the milled surface morphology of Zr-based BMG and an aluminum workpiece during the micro milling process;
2. Uncovering the influence of milling parameters on surface roughness, milling force, and phase structure.

## 2. Materials and Methods 

**Figure 1:**
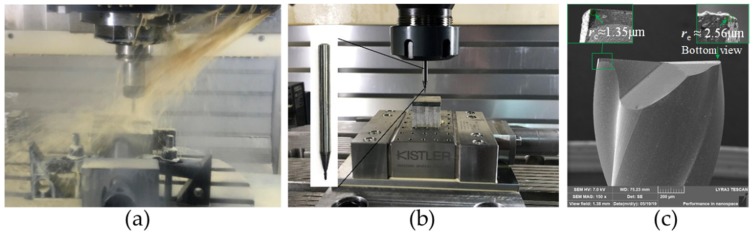
Experimental set-up for milling Zr-based bulk metallic glass (BMG). (**a**) Rough milling platform; (**b**) micro milling platform; (**c**) the two-tooth micro tool.

The micro milling experiments on a Zr_41.2_Ti_13.8_Cu_12.5_Ni_10_Be_22.5_ block (size: length 20 mm, width 20 mm, and height 3.5 mm) were conducted on a DMU 40 monoBlock CNC machine center (Bielefeld, Germany). The experimental set-up is shown in Figure 1. Rough milling was conducted to get a flatness below 1 μm on the top of the workpiece, and, in the meantime, a coolant was used to reduce the influence of heat generated in the milling process, as shown in Figure 1a. The micro milling set-up is shown in Figure 1b. All the micro milling tests were conducted in dry conditions in a full slot milling process. Two-tooth cemented carbide milling tools coated with TiAlN and CrN by an NT Tool (Takahama, Japan) were chosen in this paper. The nominal diameter of the tools was 1 mm. Rake angle and helix angles were 12° and 30°, respectively. The corner radius and cutting-edge radius were around 1.35 μm and 2.56 μm. The morphology of the micro tool is shown in Figure 1c. The aluminum workpiece Al6061 was chosen for comparison in the paper. Experimental parameters of micro milling Zr-based BMG are listed in Table 1.

A piezoelectric force dynamometer (Kistler 9119AA1, Winterthur, Switzerland) was used to measure the three mutually orthogonal cutting forces *F*x, *F*y, and *F*z. The surface roughness (Ra), three-dimensional (3D) pseudo photo, and profiles of the milled surfaces were detected using a profiler (Contour GT-X white light interferometer, Bruker, Tucson, USA). The morphology of the chips and the worn tools was observed using a scanning electron microscope (SEM, Quanta450 FEG, Hillsboro, USA). X-ray diffraction (XRD, MiniFlex 600, Austin, USA) with Cu Kα (λ = 0.154 nm) was used to detect the phase structure.

## 3. Results

### 3.1. Surface Morphology

To study the tool wear during micro milling, two new tools were utilized to mill the BMG workpiece and the aluminum workpiece. The milling parameters were as follows: rotation speed 10,000 rpm, feed rate 3 μm/tooth, axial depth of cut 30 μm, and radial depth of cut 1 mm. The total milling distance was 300 mm.

**Figure 2:**
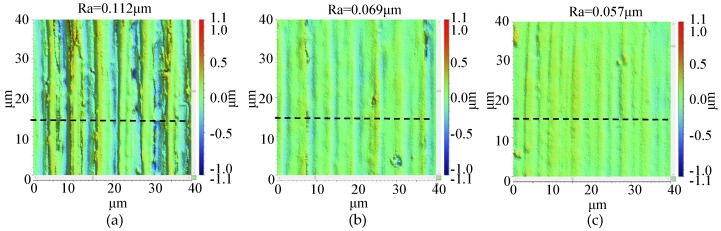
The surface morphology of the milled BMG workpiece with different milling distance. (**a**) 100 mm; (**b**) 200 mm; (**c**) 300 mm.

**Figure 3:**
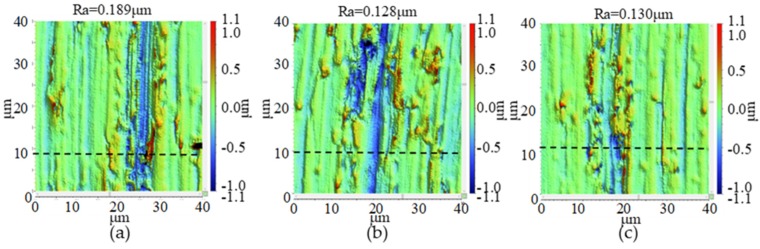
The surface morphology of the milled aluminum workpiece with different milling distance. (**a**) 100 mm; (**b**) 200 mm; (**c**) 300 mm.

The microstructures of milled BMG and aluminum surface under different milling distances are shown in Figure 2 and Figure 3, respectively. The depth of scratches on the milled BMG surface decreased as the milling distance increased, and the tool mark could be observed; on the other hand, deep and wide scratches could be observed on the milled aluminum surface, and the tool mark appeared to be irregular. In terms of surface roughness for the two materials, they both decreased as milling distance increased from 100 mm to 200 mm, and they became stable between milling distances of 200 mm and 300 mm. 

**Figure 4:**
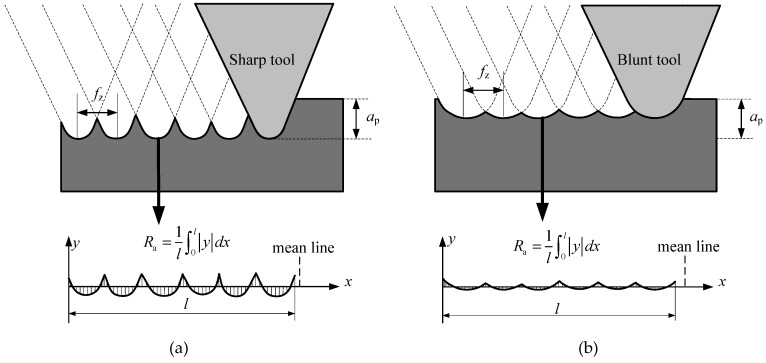
The schematic diagram of the micro milled surface. (**a**) The sharp tool corner; (**b**) the blunt tool corner.

**Figure 5:**
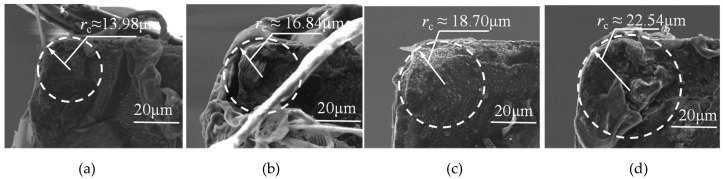
The measurement of the corner radius after a cutting distance of 300 mm. (**a**) Corner 1 for BMG; (**b**) corner 2 for BMG; (**c**) corner 1 for Al6061; (**d**) corner 2 for Al6061.

The schematic diagram of the micro milled surface is shown in Figure 4. It demonstrates that the blunt tool corner, as shown in Figure 4a, generated a larger peak-to-valley height than that of the sharp tool corner, as shown in Figure 4b, thereby reducing the surface roughness *R*_a_. The milling tool corner was assumed to be blunt; as milling distance increased, a decrease in surface roughness was witnessed in both materials. In order to confirm the hypothesis, the measurement of the corner radius after the milling process was conducted after a cutting distance of 300 mm, and the results are shown in Figure 5. The two corner radii of the worn tool for milling the BMG workpiece were 13.98 μm and 16.84 μm. The corresponding radii for milling the Al6061 were 18.70 μm and 22.54 μm. They were all much larger than that of the new milling tool.

**Figure 6:**
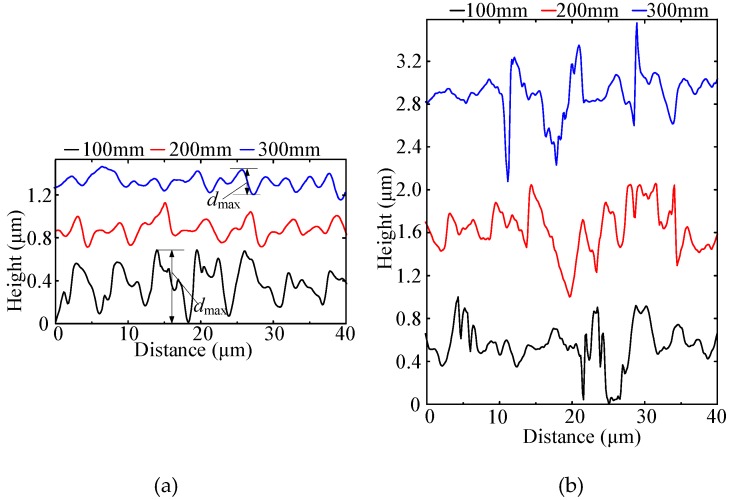
Profiles of milled BMG and aluminum workpiece. (**a**) The BMG material; (**b**) the aluminum material.

The profiles of the milled BMG and aluminum workpieces were extracted from the black dotted lines, as shown in Figure 6a,b, respectively. In Figure 6a, profile results of the BMG workpiece demonstrate that the maximum pitch depth dmax decreased as milling distance increased, from around 0.7 μm at a milling distance of 100 mm to 0.24 μm at a milling distance of 300 mm. This can be attributed to the bluntness of the milling tool from the new and sharp conditions after machining. In addition, two pitches in one circle were assumed to be generated by a milling tool with two teeth, which normally consists of a small pitch and a large pitch, as shown in Figure 6b. This is caused by the combined effect of runout and different wear conditions of the two cutting corners.

As shown in Figure 6b, wide and deep pitches appeared on the surface under all the milled distance. In terms of the pitch width, it was distributed much more randomly than that of the milled BMG surface due to the influence of smeared material. The built-up edge formation and peeling off on the rake surface could also contribute to surface formation.

In order to reveal more details of the two milled surfaces, spatial–spectral analysis was carried out using the FTT (fast Fourier transformation) approach. The spatial frequency content of the milled surface is characterized as follows [21]:

*sf*_f_ = 1/*f*_z_, (1)

**Figure 7:**
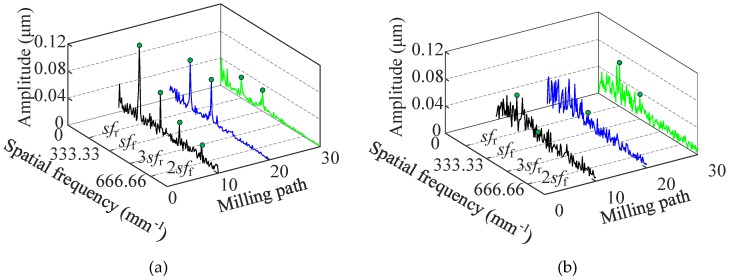
The fast Fourier transformation (FFT) spectra of the machined surfaces. (**a**) The BMG material; (**b**) the aluminum material.

*sf*_r_ = 1/(2*f*_z_), (2)

where *f*_z_ is the feed per tooth, *sf*_f_ is the spatial fundamental frequency by feed rate, and *sf*_r_ is the spatial fundamental frequency by rotation. The surface spectral is assumed to mainly consist of *sf*_f_ and *sf*_r_. Since *f*_z_ = 3 μm/tooth, *sf*_f_ and *sf*_r_ were calculated to be 333.33 mm^−1^ and 166.66 mm^−1^. In Figure 7a, the results of the milled BMG surface show that the peaks of *sf*_f_ and *sf*_r_ were quite distinct, and the amplitudes of both two peaks decreased as milling path increased. This indicates that the surface morphology generated by feed marks could be maintained well, and the depth of the pitch decreased with the increase in milling path, which is consistent with the profiles in Figure 6a. However, in Figure 7b, the results of the milled aluminum surface show that spatial–spectral properties were distributed more evenly along the spatial axis, and the *sf*_f_ and *sf*_r_ frequencies could barely be observed. This indicates that the surface morphology of milling aluminum was more chaotic, although it was also generated by the combination movement of feeding and rotation, which is consistent with the profiles in Figure 6b. 

### 3.2. Tool Wear

**Figure 8:**
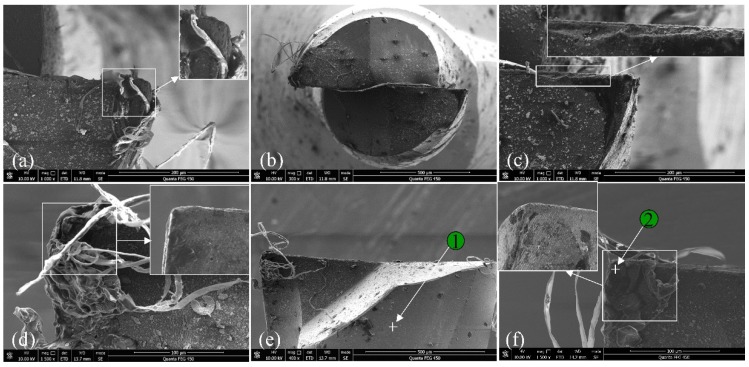
The tool microstructure after milling 300 mm for BMG workpiece. (**a**) Corner 1 bottom view; (**b**) macro bottom view; (**c**) corner 2 bottom view; (**d**) corner 1 side view; (**e**) macro side view; (**f**) corner 2 side view.

The tool microstructure after milling 300 mm for the BMG workpiece is shown in Figure 8. The tool was examined directly after the milling test without any processing. Long and continuous chips twined around the milling tool as shown in Figure 8a,b. Moreover, due to the high temperature generated in the cutting process and low conductivity of the BMG, some molten BMG material adhered to the rake surface, while the cutting edge was still exposed completely. Generally, the wear around tool tip was slight. Some rubbing wear occurred due to the friction between the workpiece and the tool, and the corner became blunt somehow, as shown in Figure 8a. In addition, tiny chipping was observed along the cutting edge adjacent to the tool tip in the bottom tooth 2, which was likely caused by the high-frequency impact during the milling process, as shown in Figure 8c. To provide a clear tool image without the twining chips, the tool was soaked in 10% NaOH solution for two hours. The tool corners after being processed are shown in Figure 8d,f, which prove that the milling tool material is an appropriate selection to cut the BMG material. Additionally, since runout exists, the wear of tooth 1 was slightly larger than that of tooth 2. 

**Figure 9:**
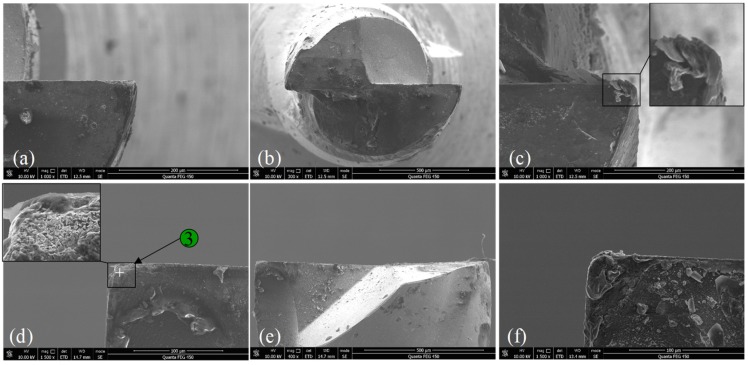
The tool microstructure after milling 300 mm for the aluminum workpiece. (**a**) Corner 1 bottom view; (**b**) macro bottom view; (**c**) corner 2 bottom view; (**d**) corner 1 side view; (**e**) macro side view; (**f**) corner 2 side view.

The tool microstructure after milling 300 mm for the aluminum workpiece is shown in Figure 9. Compared with the tool morphology in Figure 9, there were fewer twining chips. Since the aluminum is more likely to adhere and deposit on the rake surface, a built-up edge was easily formed, as shown in Figure 9c. This was the main reason for the deep and wide scratches on the milled surface shown in Figure 4b. In the meantime, many tiny particles distributed on the corner of tooth 1, as shown in Figure 9d, apparently leading to the failure of the coating.

**Figure 10:**
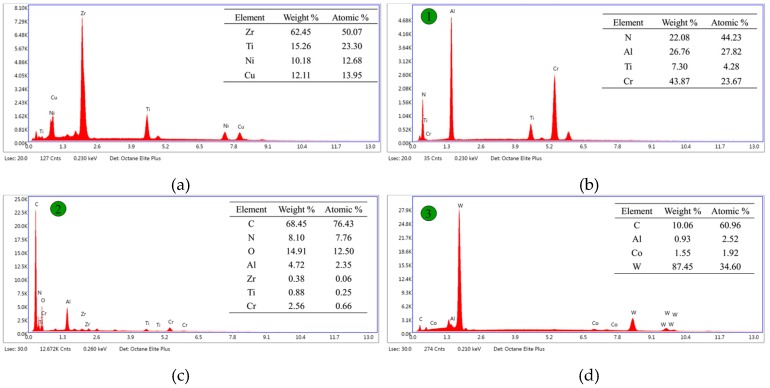
EDS (energy-dispersive spectroscopy) analysis results at different locations. (**a**) BMG workpiece; (**b**) location 1; (**c**) location 2; (**d**) location 3.

To verify coating abscission, the EDS (energy-dispersive spectroscopy) results are shown in Figure 10. Since element Be is a typical light element, it is difficult to detect in EDS, as shown in Figure 10a. The sole coating EDS results indicate that Ti, Al, N, and Cr were the main elements of the coating, as shown in Figure 10b. After milling BMG, all coating elements could be still detected on the rake surface, although their weight percentage decreased dramatically. Coating abscission occurred due to the detection of the C element. Moreover, element O was found at location 2 shown in Figure 10c, which indicates that there was an oxidation phenomenon in the milling BMG process, while it could not be detected on the rake surface after milling the aluminum workpiece, as shown in Figure 10d. This may be attributed to the significant difference of conductivity between the two materials, i.e., 4 W/(m·K) for BMG and 167 W/(m·K) for aluminum [7]. Since the heat is more difficult to dissipate in milling BMG, the temperature is assumed to be much higher than that in milling aluminum, thereby facilitating the oxidation phenomenon. After milling aluminum, the major compositions were W, C, and Co at the rake surface, as shown in Figure 10d, and these are the main elements of cemented carbide. This proves that the coating peeled off severely. 

### 3.3. Chip Morphology

**Figure 11:**
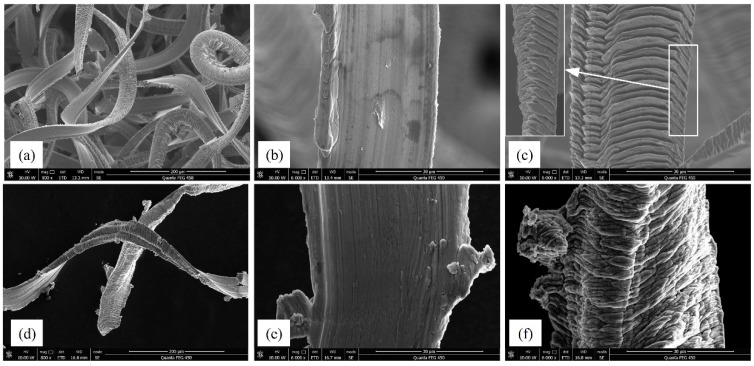
Chip morphology of the BMG and aluminum workpiece. (**a**) BMG macro view; (**b**) BMG back surface; (**c**) BMG free surface; (**d**) aluminum macro view; (**e**) aluminum back surface; (**f**) aluminum free surface.

The typical chip morphologies of the two materials are shown in Figure 11. The macro images in Figure 11a,d show that both materials experienced ductile mode cutting during the milling process. In terms of the back surface, it was relatively smooth for BMG chips, indicating that the formation of the built-up edge was negligible, while deep marks along the cutting direction could be observed on the aluminum chip due to the influence of built-up edges. As for the free surface, prominent shear lamellas separated by regions of shear localization were observed on the BMG chips, as shown in Figure 11c. The enlarged side view indicates that the shear lamella was only held together by a very thin layer at the back of the chip. The main reason is that no-slip systems like crystalline metals exist in the BMG material, and the slipping is only controlled by the maximum shear stress, which made it easier to form the segment chip [4]. As for the aluminum chip, wavy slip lines of irregular period and small amplitude were observed with no serration, as shown in Figure 11f.

### 3.4. Surface Roughness

Since the tool wear was only slight after milling 300 mm, and low surface roughness could be obtained as discussed in Section 3.1 and Section 3.2, two new milling tools were utilized for micro milling BMG and the aluminum workpiece. To eliminate the influence of tool wear and only focus on the influence of milling parameters on the surface roughness, all surface roughness curves were plotted as a deviation pattern based on a base point at a milling speed of 10,000 rpm, axial depth of cut of 30 μm, and feed rate of 3 μm/z. 

**Figure 12:**
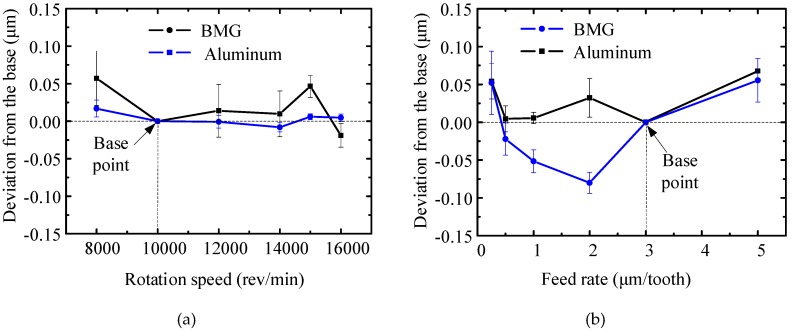

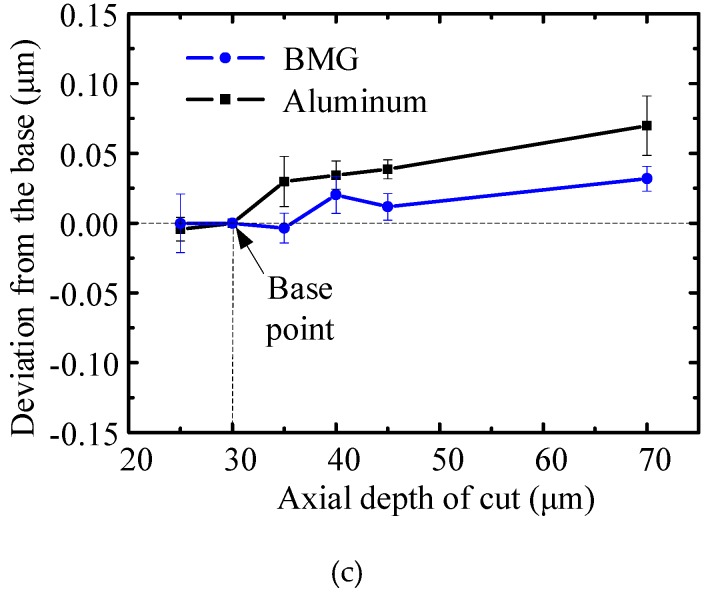
The influence of milling parameters on surface roughness. (**a**) The influence of rotation speed; (**b**) the influence of feed rate; (**c**) the influence of axial depth of cut.

The influence of rotation speed on surface roughness is shown in Figure 12a. Its influence on milled surface roughness was insignificant in both cases. Since there are usually deep marks left randomly on the milled surface, the curve of the aluminum workpiece experienced more fluctuations, while the fluctuation range of the BMG curve was 0.05 μm. Fujita et al. [9] also found a similar trend when turning Zr-based and Pd-based BMG.

**Figure 13:**
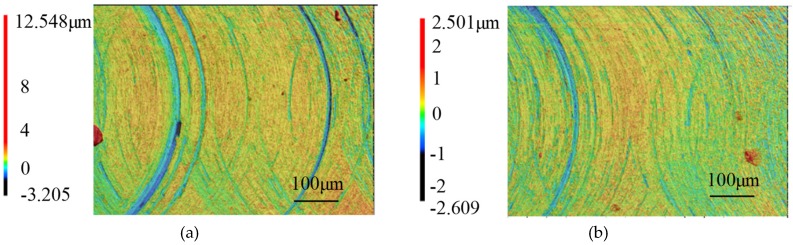
The surface morphology for aluminum material. (**a**) Feed rate of 2 μm/tooth; (**b**) feed rate of 3 μm/tooth.

The influence of feed rate on surface roughness is shown in Figure 12b. For the aluminum curve, an obvious size effect was witnessed when the feed rate was below 1 μm/tooth. In the meantime, the curve was assumed to increase as feed rate increased, while there was a reverse trend at 2 μm/tooth and 3 μm/tooth. The corresponding surface morphology is shown in Figure 13a,b, respectively. Deep marks can be observed on the milled surface at a feed rate of 2 μm/tooth as shown in Figure 13a, and the surface roughness increased sharply, while the marks generated on the milled surface at a feed rate of 3 μm/tooth is relatively shallow, and the roughness value decreased obviously. In terms of the BMG curve, an obvious size effect was witnessed when the feed rate was below 2 μm/tooth, and the surface roughness decreased dramatically as the feed rate increased in the range. As the feed rate increased further, the surface roughness increased due to the morphology generated by the feed marks. The optimal feed rate was around 2 μm/tooth.

The influence of axial DOC on surface roughness is shown in Figure 12c. Both roughness curves increased gradually with the increase of axial DOC. According to the proposed surface roughness model in the milling process [22], axial DOC is not the main factor influencing the surface roughness. This may be attributed to the fact that micro milling in high axial DOC conditions could generate high milling force and high deformation, which deteriorates the surface quality. The variance of the aluminum curve was more obvious than that of the BMG curve. Furthermore, although the actual surface roughness is not shown in Figure 12, it is worth pointing out that the roughness values of the milled BMG surface were all lower than those of the milled aluminum surface under the same milling conditions, and a precise finishing level (*R*_a_ = 0.13–0.2 μm) could be obtained for the milled BMG surface. 

### 3.5. Milling Force

**Figure 14:**
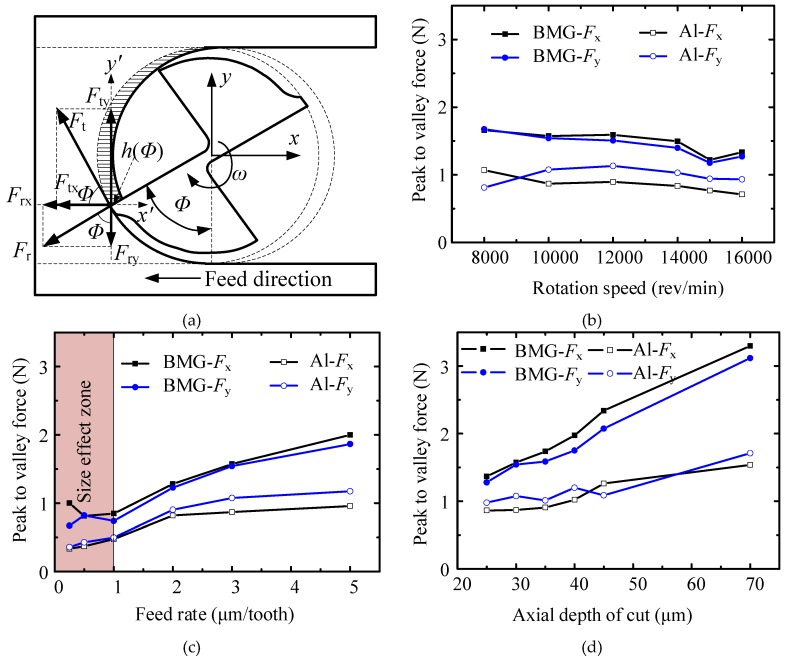
The influence of milling parameters on milling force. (**a**) The schematic diagram of the milling process; (**b**) the influence of rotation speed; (**c**) the influence of feed rate; (**d**) the influence of axial depth of cut.

The measured cutting forces were low filtered with a cutoff of 800 Hz to compensate for the distortion of cutting forces caused by the dynamometer dynamics [16]. The force value was obtained by averaging peak-to-valley forces in 10 cycles for quantitatively study. The schematic diagram of the milling process is shown in Figure 14a. *F*_x_ is parallel to the feed direction, and *F*_y_ is perpendicular to the feed direction. The influence of milling parameters on milling force is shown in Figure 14b–d. 

The influence of rotation speed on the milling force is shown in Figure 14b. Due to the different ultimate tensile strength [7] (1900 MPa for BMG and 300 MPa for aluminum), the milling force curves of the BMG material in both *x-* and *y*-directions were much larger than those of aluminum material. The influence of rotation speed on milling force for the two materials was insignificant, although a slight decrease was witnessed as the rotation speed increased over 14,000 rpm due to the softening effect of the material with increasing temperature.

The influence of the feed rate on the milling force is shown in Figure 14c. Since a high feed rate can lead to a large cross-sectional cutting area, the milling force curves for both materials experienced an obvious increase as feed rate increased. It is worth pointing out that both curves experienced a nonlinear trend as the feed rate decreased below 1 μm/tooth. This phenomenon can be mainly attributed to the effect of minimum uncut depth (MUD). According to the conclusion in Reference [23], the minimum chip thickness for the majority of crystalline metal materials reported in the literature is around 0.05–0.4 times the cutting-edge radius. Although few papers reported the MUD for BMG material, the MUD of BMG is assumed to be around the same range as that of crystalline metal materials, which is around 0.125–1 μm. When the feed rate approaches the MUD, the main cutting force is generated by ploughing instead of shearing, which leads to an increase in cutting force.

The influence of axial DOC on milling force is shown in Figure 14d. The milling force curves of the two materials both increased as axial DOC increased due to the increase in cross-sectional cutting area. Moreover, the slope factor of the BMG milling force curve was much larger than that of the aluminum milling force curve.

### 3.6. XRD Analysis 

**Figure 15:**
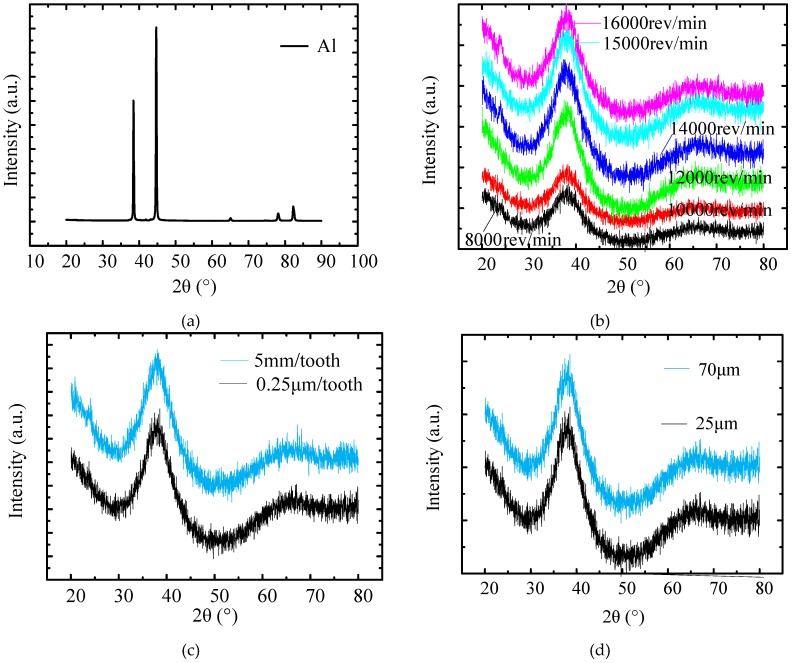
The influence of milling parameters on phase structure. (**a**) X-ray diffraction (XRD) result from a ZL101A workpiece; (**b**) XRD results under different rotation speeds; (**c**) XRD results under different feed rates; (**d**) XRD results under different depths of cut (DOCs).

The XRD result of a typical crystalline material aluminum is shown in Figure 15a. There are several clear and sharp peaks. The XRD results of the milled BMG workpiece under different rotation speeds, feed rates, and DOCs are shown in Figure 15b–d, respectively. In Figure 15b, there is a broad peak at around 2θ between 30° and 50° on each curve, with a few sharp peaks, which indicates that the BMG workpiece still kept the amorphous structure in all milled surfaces under different rotation speeds.

Since the influence of the milling parameters on the amorphous structure is supposed to be linear, the XRD results under only the minimum and maximum feed rates and DOCs are demonstrated in Figure 15c,d. One broad peak can be witnessed in each curve, while some tiny sharp peaks appeared on the peak in Figure 15d. Therefore, the XRD results indicate that all milled BMG surfaces were still dominated by the amorphous structure under current milling conditions. Although Maroju et al. [15] found that full crystallization occurred in high-speed milling of Zr-based bulk metallic glass, their cutting speed was around 50,000–60,000 rpm, the tool diameter was 3.175 mm, the axial depth of cut was 2 mm, and the radial depth of cut was 0.45 mm. These values are all much larger than the parameters in this paper. In other words, the increase in temperature in the material deformation zone was not high enough to cause crystallization in the research.

## 4. Discussion

### 4.1. Comparison between Zr-Based BMG and Stainless Steel

In addition to their excellent mechanical properties, Zr-based BMGs have better corrosion and wear resistance and biocompatibility than conventional metal materials, which makes them promising materials in biomedical applications, e.g., bone fracture fixation and hip arthroplasty components [24]. Since 316L stainless steel is currently still the most used alloy in all implants ranging from cardiovascular to otorhinolaryngology [25], the main mechanical properties of the two materials are listed in Table 2 for comparison. 

According to Table 2, Zr-based BMG is superior in terms of almost all mechanical properties except for Young’s modulus. However, it is worth pointing out that a low modulus comparable to the bones is critical for avoiding stress shielding [26]. The Young’s modulus of cortical bone is 3–50 MPa, and it is closer to the value of Zr-based BMG, which indicates that the Zr-based BMG is more suitable in this aspect as well. 

**Figure 16:**
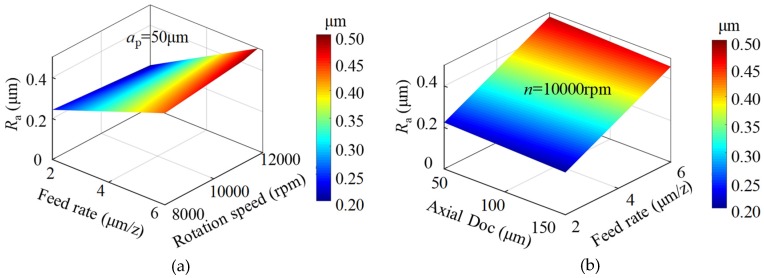

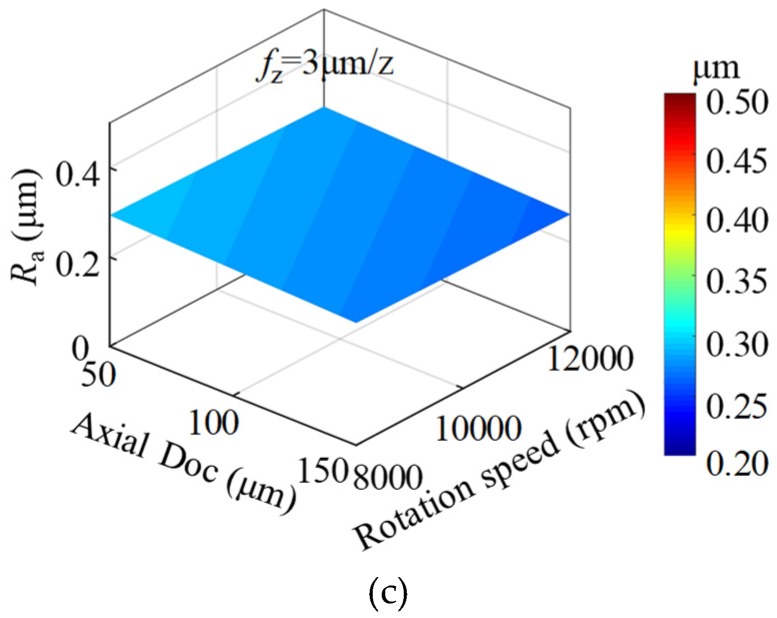
The parameter mutual effects on milled surface roughness of AISI304 stainless steel. (**a**) Feed rate and rotation speed; (**b**) axial DOC and feed rate; (**c**) axial DOC and rotation speed.

In terms of the milled surface roughness, Kuram and Ozcelik [27] conducted micro milling experiments on stainless steel with similar milling parameters. Although the nominal diameter of the TiAlN-coated cemented carbide tool was 800 μm, the results are assumed to be comparable to the results in the paper. Based on their established surface roughness model, the parameter mutual effects on surface roughness during micro milling stainless steel are shown in Figure 16. Under the parameter conditions, it is obvious that the surface roughness of milled stainless steel was between 0.25 and 0.5 μm, which is much higher than that in this paper (below 0.2 μm). Therefore, it is easier to obtain good surface roughness when micro milling BMG than when micro milling stainless steel.

### 4.2. A Potential Application: Fabricating the Mold for Microfluidic Polymeric Devices

Due to its excellent mechanical properties, BMGs were utilized as tool inserts for the microinjection molding of polymeric microfluidic devices [28]. However, the microstructure of BMGs is normally patterned on a Si wafer using standard lithography and deep reactive ion etching (DRIE) techniques [28], which is time-consuming and expensive. In the meantime, it is much easier to obtain the microstructure by micro milling. In order to demonstrate the practical application of micro milling on the BMG material, the fabrication process of a typical microstructure on the microfluidic chip is demonstrated below.

**Figure 17:**
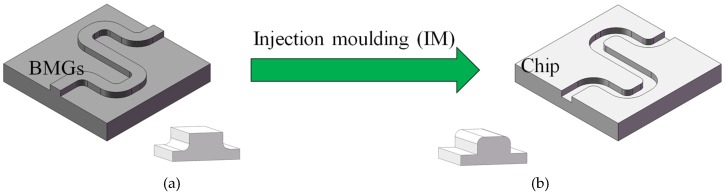
The inappropriate process for fabricating a microstructure on the chip. (**a**) The micro milled mold; (**b**) the obtained microstructure.

Normally, the positive shape on the mold for IM (Injection moulding) is as shown in Figure 17a. If it is obtained by micro milling, the majority of the material on the surface is supposedly removed by the milling process, which is time-consuming. In addition, due to the existence of the corner radius of the milling tool, it is impossible to obtain a sharp inner corner on the chip, as shown in the bottom image of Figure 17b. However, some applications require sharp corners, i.e., geometric capillary flow stops [29].

**Figure 18:**
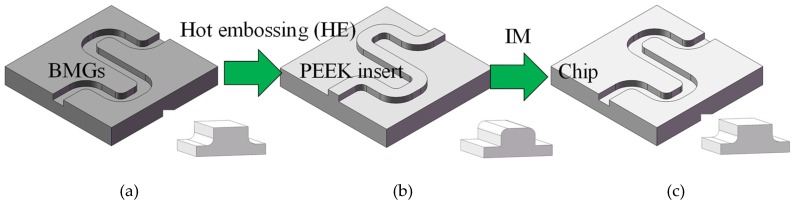
The optimized process for fabricating a microstructure on the chip. (**a**) The micro milled mold; (**b**) the PEEK insert; (**c**) the obtained microstructure.

To tackle the mentioned disadvantages, the optimized process is shown in Figure 18. Firstly, the same microstructure is micro milled on the BMG material, and the negative microstructure is achieved; then, the high temperature polymer insert PEEK (Polyetheretherketone) is fabricated by HE (Hot embossing); finally, the PEEK inset can be used for IM. The approach was proven to be effective in Reference [30].

## 5. Conclusions

In this paper, micro milling tests were conducted to study the machinability and surface integrity of Zr_41.2_Ti_13.8_Cu_12.5_Ni_10_Be_22.5_ bulk metallic glass. The main results could be concluded as follows:

1. The coated cemented tool was still in stable wear stage after milling Zr-based BMG for 300 mm, and the surface roughness Ra could be maintained around 0.06 μm. The tool experienced slight wear with small chipping and rubbing wear during milling the Zr-based BMG, while the built-up edge and coating peeling off occurred severely when milling Al6061.
2. As for the BMG material, the influence of rotation speed on surface roughness was insignificant, while surface roughness increased gradually with the increase in axial DOC. Surface roughness decreased with the reduction of feed rate, then increased sharply when the feed rate approached 2μm/tooth due to the size effect. All surface roughness values of milled BMG surfaces were much lower than those of milled aluminum surfaces with the same milling parameters.
3. As for the BMG material, milling force decreased slightly with rotation speed, while it increased rapidly with axial DOC. Milling force decreased linearly with the decrease in feed rate, while a nonlinear phenomenon occurred when the feed rate was below 1 μm/tooth. All milling forces of the BMG workpiece were higher than those of the aluminum workpiece with the same milling parameters.
4. The X-ray diffraction testing results indicated that, under the milling parameters in this paper, all milled surfaces could still maintain the amorphous structure in dry machining conditions.

## Figures and Tables

**Table 1:** Experimental parameters of micro milling Zr-based bulk metallic glass (BMG).

Single Factor	Rotation Speed (rpm)	Cutting Speed (m/min)	Feed Rate (μm/tooth)	Depth of Cut (μm)
Rotation speed	8000, 10,000, 12,000, 14,000, 15,000, 16,000	25, 31, 38, 44, 47, 50	3	30
Feed rate	10,000	31	0.25, 0.5, 1, 2, 3, 5	30
Axial depth of cut	10,000	31	3	25, 30, 35, 40, 45, 70

**Table 2:** Comparison of two common biomaterials in terms of mechanical properties [25].

Mechanical Properties	316L	Zr-Based BMG
Tensile yield strength (MPa)	190–690	1900
Elastic strain limit (%)	0.34	2.0–2.2
Young’s modulus (GPa)	193–210	90
Hardness (Vickers)	365	590
Fatigue limit at 10^7^ cycles (MPa)	200–800	910
Density (g/cm^3^)	7.9	5.9
